# Memoirs of the Life of the Late Mr. John Greenwood

**Published:** 1840-05

**Authors:** E. Bryan


THE AMERICAN JOURNAL

OF

### DENTAL SCIENCE.

VOL. I.—NO. V.

[May, 1840.]

### MEMOIRS

OF THE LIFE OF THE LATE MR. JOHN GREENWOOD,

*Mechanical and Surgeon Dentist, of New-York City:—Compiled by*

E. BRYAN.—(CONTINUED.)

As soon as my father found out that I was among what they called the *Rebels*, he went to Governor Gage and obtained permission for my mother to go to the American camp, in order to procure a substitute for me, and then to return with me to Boston. She arrived at our camp the day before the battle of Bunker Hill, but was unable to accomplish her purpose and not permitted to return to Boston until the expiration of six weeks, when, on the arrival of General Washington, she made personal application to him for permission to return, which was immediately granted, though against the wishes of many of the officers. She was conducted to the British line by one of our sergeants, and upon being carried to the commanding officer of the British Fort, Major Small, he treated her with the greatest politeness, and conducted her himself, to my father's house in Boston.

After the battle of Bunker Hill, nothing material occurred between us and the British, for some time; they would occasionally amuse themselves by throwing their bombs into our fort, but with little or no injury; and we, on the other hand, by firing at their guard-house, over Roxbury neck.

General Washington, having many spies in Boston, was readily informed of all that was passing in that city. It happened, on one occasion, that he was notified of the intention of the British officers to bring out a new dramatic entertainment, to be performed by them on a certain night, at the Theatre, which was entitled " The Blockade of Boston," and intended to ridicule the *Yankees*, as they termed us. When General Putnam heard this, he determined, by way of retaliation, to set fire to the buildings in the vicinity of the British fort, at Bunker Hill, and near the old causeway, belonging to Charlestown Mills. Here were twelve or fourteen houses, occupied by sutlers, mechanics, soldiers' wives and stragglers, belonging to the British army.

Accordingly, General Putnam having procured large quantities of chips, he caused them to be dipped in turpentine and brimstone, and then loading thirty or forty men with these inflammable materials, done up in convenient bundles, he proceeded, at the head of two hundred men, between nine and ten o'clock at night, to march, silently and cautiously, to the site of our intended attack.

Being at this time fife-major, it was optional with me to go or not, as I chose, but being curious to know what was to be done, I determined to accompany them, though my fife was not required upon this occasion, as we had strict orders to proceed without the least noise. We passed over the causeway undiscovered, and were thus enabled to surprise the different sentries—set fire to the houses, and make prisoners of the inmates, before the British could be aware of our presence, which, however, they soon found out by the blaze of their houses, when they commenced firing from their fort; but we were quickly out of their reach, and I did not learn that we lost as much as one man in this affair.

This conflagration caused no small alarm in Boston, where it was distinctly seen, and at the moment when one of the performers at the theatre, was personating a *Yankee* Soldier, dressed as a tailor, with his shears projecting out of his pocket and his measures hanging over his shoulders, when a sergeant ran hastily upon the stage to give the alarm, and exclaimed, at the top of his voice, " to arms ! to arms ! gentlemen ! ! the Rebels are upon us ! !" but the audience, supposing him to be acting his part in the play, instead of taking the alarm, loudly applauded him for the fidelity of his acting, and it was some time before he could make them believe that the part he *acted* was no mummery ; when, however, they understood him, the performance was instantly suspended and they rushed pell-mell out of the house. My father and mother were in the theatre that night and witnessed the whole proceedings.

In the month of April or May, 1776, the British evacuated their fort at Bunker Hill and soon after set sail from Boston for Halifax, Nova-Scotia. About that time, our regiment, consisting of five hundred tolerably well disciplined men, received orders to march to New-York, and thence to Canada. On our arrival in Montreal, we took up our quarters in the large stone Barracks, adjoining the north gate of the city. In a few days many of our men were attacked with the Small Pox, and were removed to the hospital. The main division of our army was encamped at a place called " Three Rivers." One regiment, commanded by Colonel Beetle, was stationed up the St. Lawrence River, at a place called " The Cedars," about thirty-five miles from Montreal ; they were about six or seven hundred men strong, entrenched in a small fort, but so completely invested by the enemy that they were unable to obtain any supplies of provisions. In this extremity, Colonel Beetle contrived to send an express to General Arnold, who commanded at Montreal, informing him of his destitute condition, urging an immediate supply of provisions and a re-inforcement of men : but before a re-inforcement could come to his relief, he was compelled to surrender to the mercy of the Indians, who amounted to thirteen hundred warriors, besides a company of Canadian soldiers, and about sixty British troops, with two brass cannons. General Arnold detached two hundred men from our regiment, under command of Major Sherbourn, accompanied by the captain of my company, whose name was Bliss, also by Lieutenant Edward Comstock, and with three waggons loaded with provisions ordered to hasten to the relief of Colonel Beetle. After marching thirty miles to Fort Ann, they had to cross the St. Lawrence, and then travel three or four miles from the opposite shore, before they arrived at " The Cedars." The Indians seeing them approach, suffered them to proceed unmolested, until they arrived within two miles of " The Cedars," when, having secreted themselves along the road they had to pass, in the woods on each side—the Indians suddenly sprung up, and uttering their horrid war-whoop, poured into their ranks a most destructive fire. They bravely fought the Indians for two hours and then being overwhelmed by numbers had no alternative but to surrender themselves prisoners of war to the savages. When Major Sherbourn crossed the river, he left some of his men in charge of the boats, which the Indians perceiving, sent some of their party, by a circuitous route to the spot, in order to destroy the boats and take the men prisoners, which they effectually performed, so as to cut off all chance of escaping out of their merciless hands.

The Indians killed, in this engagement, upwards of sixty of our men and wounded many more,—and in return, many of the Indians bit the dust, and among them, several of their chiefs, which so enraged the Indians that they stripped all the clothes off our men, that were worth taking, and then butchered all our wounded, that they might not impede their travelling. The manner in which they despatched our wounded, was by knocking them on the head with their tomahawks, and then, as is their custom, taking off their scalps, which they carried with them as trophies of war. Amid hideous yells, they drove their prisoners forward to an old stone church, in the vicinity of " The Cedars," which they had converted into a prison, in which they had secured Colonel Beetle and his men, who were in a most distressing condition for want of provisions, and the Indians, being themselves short of supplies, had killed all the cattle they could find for a considerable distance round, excepting a cow which belonged to the Catholic Priest, which they were forbidden to touch. At length, however, they lost all respect for the priest or his cow, and knocking her on the head, they cut off her flesh while yet alive with their scalping knives, without even skinning her, and soon devoured both the flesh and the entrails. So much cruelty, however, did the Indians manifest towards their prisoners, that if it had not been for the British troops, who restrained them, they would have burnt or otherwise have murdered every one of them.

Elated with their success, the Indians now began their march towards Montreal, with the intention of attacking us. They compelled their prisoners to accompany them; but as many of the prisoners were without any clothing, destitute of provisions and suffering from the flux, the fever, and exposure during the night, there were many unable to travel with the rest; these were from time to time tomahawked and scalped.

General Arnold, who had intimation of their approach, mustered all the men that were fit for service, to go out and attack them; but as two thirds of our troops were in the hospital, suffering with the small pox, he was unable, after leaving enough to protect the city, to call out more than five hundred troops. At the head of these men he marched out of the city. I was with the advance guard. We had not proceeded more than two miles before we saw three Indians in the road, coming towards us—the moment they saw us, they gave the war-whoop, threw down their guns and blankets, ran into the woods and disappeared in an instant. Our guide was a deserter from the British, who had belonged, to what is called the "Rangers." Our general ordered him to cross the woods for the purpose of reconnoitering the enemy, and I was instructed to accompany him. He was armed only with a tomahawk—I, with a small fuzee and a sword. Being alone with him in the woods, I began to be apprehensive that he might show me *foul play*, as I never liked the idea of trusting too much to deserters, who, however they may apparently favor your cause, still they have been traitors to *their* country, and *may* prove traitors to you, when a convenient opportunity affords. I, therefore, kept myself on my guard, by walking at a little distance from him, so as to be ready in case he should make an attack, and if he should do so, I thought I should be a pretty good match for him, as my sword was sharp and my gun loaded; however, I had no reason to complain of his behaviour, as the event will show. We proceeded nearly ten miles, without meeting any one; at length we came to a fence, when we perceived a small house at a short distance. He informed me that he was acquainted with the people of the house and wished to go there alone, as my presence would excite suspicion, on account of my uniform, which was a blue coat, turned up with buff and trimmed with silver lace. I told him I saw the propriety of taking every precaution to avoid exciting suspicion and readily agreed to wait by the fence until he returned, which he did in a few minutes, and then desired me to go back with him to the house, which I did. I found the inmates to consist of an aged Canadian woman, about seventy-five, as I should judge—a Canadian man, fifty, and his wife, about thirty, who was a squaw that he had purchased of the Indians. It was now beginning to get dark and we had not been in the house more than fifteen minutes, when we heard the hideous war-whoop of the Indians. We had scarcely time to secrete ourselves under the bed, when the room was filled with Indians, making the most frightful noises and incessant screaming. I now began to feel that my situation was indeed a most perilous one, as, if they had discovered us, instant death would have been our certain lot; but luckily for us, the Indians were in such confusion that they thought more of their own safety, than of searching the house for spies—as we afterwards learned,that by cutting through the woods we had got ahead of them, and that General Arnold had come up with them, and was pursuing them so closely, that they were retreating in the utmost precipitation and disorder. In about an hour, all the Indians had passed by, and we gladly came forth from our hiding place. My companion wishing to disguise himself, exchanged some of his clothing with his Canadian friend, which having put on, he said "we will now follow the Indians and see what they are about:" we did not, however, go on the public road, but took the precaution to cut across the lots, when, after travelling about three miles, we came up with them at Fort Ann, where was a small town or settlement; outside of which, and on the inside of a fence, he desired me to wait his return, as he wished to enter the town, disguised as he was, and observe their movements. The signal he was to give me on his return, was, that without speaking, he would come along, beside the fence, with one hand touching it all the way, until he touched me, and then I might be sure it was him. I should observe, that this fence, like many others in this country, was constructed of logs set upright and driven into the ground close to one another, so that as I was on the inside of this fence, those who passed on the road could not discover me, especially as it was dark; nevertheless, I began to think myself in a very critical situation; what, thought I, will be my lot,if my companion should be detected, or any mishap should prevent his returning. I never could have found my way back, alone, and should have had to remain there till morning when I should, at least, have been taken prisoner. I was within hearing of the ferocious savages ; every minute seemed an hour. I heard some of them walking by me in the road, and I began to fancy that they were in search of me. In this situation of anxiety and suspense, I remained for about an hour, when my comrade returned and touched me in the manner agreed upon. It made my heart jump. "Come along," says he, "the Indians are crossing the river as fast as they can, and will be all over before our troops can come up with them; we must go and inform the general." Accordingly, with all haste, we took our route back, over fences and fields, for about two miles, when we fell in with our troops and informed the general of the situation of the Indians. It was now nearly day-light. The troops were immediately ordered to pursue them, for which purpose they embarked on board of the Batteaux, together with two hundred Indians belonging to a tribe hostile to those we were in pursuit of, and who had opportunely joined us at this place. General Arnold was rowed by two of them in one of their thin bark canoes. The current being very rapid, and we going against the stream, we did not reach Fort Ann till about sun-set. We perceived a man in the river, making towards our boat—we took him in, he proved to be one of our men, who had been taken prisoner by the Indians, and offered to conduct us to where they were.

We pushed on until we got within musket shot of the opposite shore, where the river is very wide. It was now within half an hour of sunset, when a numerous body of Indians suddenly made their appearance, from behind the trees which lined the shore; they showered upon us their bullets, as thick as hail-stones, so that we were unable to make good our landing, and it being now sun-set, General Arnold gave orders to retreat to the opposite side of the river. The British troops had also drawn their two field pieces to the bank of the river, which made sad havoc among our Indians' canoes. As soon as we had disembarked on the opposite shore, our general ordered us to make a great many fires to induce the enemy to suppose that we were very numerous, and that they might consequently expect us to renew the attack in the morning. This *ruse de guerre*, had the desired effect: about midnight a flag of truce arrived from the British, offering to give up to us all the prisoners that had been taken from us by the Indians, on condition, that they should not be allowed to serve against them, for the term of seven months, and that certain of our officers should be given up to them as hostages for the faithful performance of the terms stipulated; to this we readily consented, and the next morning they returned us all the prisoners, and took back with them some of our officers—but I cannot say how many there were; but among them was Captain Bliss, of the company to which I belonged.

We now ascertained that the British were advancing upon us, on both sides of the river from Quebec; and as many of our men were suffering from the small pox, our general gave orders that we should immediately return to Montreal, in order to make preparations for evacuating that city before the arrival of the British. On the second day after our return to Montreal, late in the afternoon, the order was given, "Retreat! retreat! the British are upon us!!" Down we scampered to the boats, in the greatest confusion, leaving our camp equipage, cooking utensils, and even our bread cooking in the oven, regardless of every thing, but our own immediate self preservation. We did not, however, forget our sick comrades who were assisted from the hospital down to the boats. When we were about half way across the river, the rain began to pour down in torrents, and it being excessively dark, it was difficult to land on the opposite side. We were all pretty well soaked to the skin, and many of our poor fellows, who were suffering at the time with the small pox, died in consequence of their exposure and the hardships they experienced.

As we landed, every one seemed disposed to take care of himself as well as he could, and the sick where left to shift for themselves. I found the way to an old barn, which I entered and soon ascertained that others had discovered the same retreat. I laid down, thinking to have a tolerable good night's rest, but I had hardly begun to get warm and comfortable, when some of our officers pushed open the door and knowing pretty well that some of us had ensconced ourselves in those comfortable quarters, they began to sing out lustily, "Turn out! turn out!! d——n you, turn out and march to join the army. Turn out! or we will fire upon you!!" Knowing it was too dark for them to see us, we made no answer at first, but at length we *woke up*, and told them we were ready to march, half way up to our knees in mud, a distance of three miles, where General Arnold was mustering his scattered forces. Seeing a wind-mill, near where I stood, I slipt into it unobserved, and going into the upper apartment, I laid myself down and slept comfortably until I was awakened at day break, by the drums beating to arms, when I re-joined my regiment, without being questioned by the officers. General Arnold called together the catholic priests and friars in the town or settlement where we were, and threatened them, that if they did not immediately procure a sufficient number of Carts and waggons to transport our baggage and our sick to La Prairie, he would set fire to the town.

Thus urged, a sufficient number of waggons were soon put in requisition for our purpose, and we set out on our march. Our road lay beside the river, so that we could plainly see the city of Montreal, and that there appeared to be a great many Britsh troops there, who were easily distinguished by their red coats.

We shortly passed a bridge, which we set on fire to prevent being followed; and presently after, we saw along the shore, a number of our boats which had landed the troops the night previous, for so rapid was the current, that many were carried a considerable distance down the stream before they were able to effect a landing; these were the boats we now met with, and among them, those which had contained the poor fellows who were sick with the small pox; and what a shocking sight now met our eyes! we beheld the dead bodies of these, our late unfortunate comrades, strewed along the shore! some had struggled, perhaps, with just sufficient strength to reach the land, there to perish with fatigue and exhaustion, probably, overwhelmed by their exertions, exposure and disease, had either been capsized or fallen into the water, and washed ashore, while others, who had safely landed by the assistance of those able to help them, were left during the night to shift for themselves, exposed to a heavy rain, without any shelter. General Arnold, in anticipation of our retreat, had previously caused considerable stores to be landed on this side the river, consisting chiefly of wines, butter, raisins, &c. &c., but we were obliged to leave the greater part of these desirable things behind us. The boats were ordered to be destroyed to prevent them from falling into the hands of the British. We made but a short stay at La Prairie, and hurried on to St. John's at the head of Lake Champlain; there we remained a few days to muster our scattered troops, and recruit their strength.

Our general having now procured a number of open boats, we all embarked for Ticonderoga. Being short of provisions, and without camp kettles or other cooking utensils, it may be supposed that our situation was far from being agreeable. Our daily rations consisted of only a pint of flour and a quarter of a pound of pork for each man, and every day, at noon, we used to land for the purpose of cooking our food. For want of vessels in which to mix our flour, we made and baked our cakes on pieces of the bark of the trees, but such cooking was any thing but tempting, especially to the sick, who fared no better than the rest. For myself, I did not fare quite so bad as my comrades, being a favorite with the officers, besides, being fife-major, I was not called upon to row, or do the least of what is called *Fatigue;* I also had my regular rest every night. I employed much of my time in playing lively tunes on my fife, for the purpose of cheering up my comrades, especially the sick, and took every opportunity to assist them and make them comfortable, as far as was in my power.

At length we arrived at Ticonderoga, where we found three regiments of troops from New England; they, with ourselves, were ordered to proceed immediately to Mount Independence, a high mountain, opposite Ticonderoga, which was covered with trees, very rocky, and abounding with snakes of all descriptions, especially black and rattle snakes. Here we made our encampment, and as there was plenty of cattle to be had, we were served every day with plenty of fresh meat, but being unable to procure any salt, many of our men were carried off by the flux, so that what with those we lost in Canada by small pox, and those who died at this station, we had but about one hundred men left out of a regiment of five hundred. Our general had contrived to procure three or four floating batteries; a schooner, a sloop and other small crafts, amounting in all to thirteen vessels, which were intended for our protection on the river. This we called our Mosquito Fleet. We had been here between two and three months, without any thing to disturb us, when a British ship hove in sight; she carried twenty brass six pounders, and was accompanied by a number of gun boats, well manned. Arnold commanded our mosquito fleet, but being overpowered by numbers, was forced to retreat.

All our troops were now ordered to march to Albany; during which, we suffered much for the want of tents to shelter us from the snow and rain, and being myself, now on the sick list, with the fever and ague, I began to feel the hardships I had to endure as almost intolerable. No sooner had we arrived at Albany, than we were put on board vessels with orders to proceed to Esopus; from thence we marched to Newtown, Pennsylvania. Two days after, we received orders to march to Trenton, New Jersey. The sun was nearly down when we crossed the river Delaware, and amidst a tremendous storm of rain, hail, and snow by turns; we proceeded at a slow pace, travelling all night, and between seven and eight in the morning, came in sight of Trenton. The first thing that occurred between us and the enemy, was the firing of a six pound cannon at us, which struck the fore horse that was drawing a three pound cannon, (the only one we had.) The weather being stormy and hazy, we could not see far ahead; we marched on, when suddenly, within two hundred yards of us, we discovered a body of between three and four hundred Hessians, commanded by Colonel Roll; they were paraded two deep in a line, and their commander on horse-back on their right; they made a full fire right at us, but I did not see any one fall. Our major (Sherbourn,) ordered us to fall back, about three yards, to pull off our packs and pile them by the side of the road. "Now my brave boys," says he, "pass the word through the ranks, that he who is afraid to follow me, may stay behind to take care of the packs." Not a man offered to leave the ranks, and we lost all our packs, for we did not return that way.

Having been out all night in the storm, our guns and powder were wet; orders were therefore given to charge bayonets; but of these we were very deficient. When we arrived within pistol-shot of the Hessians, they fired against us—we dodged, and they did not hit a man. Before they had time to re-load, we were within three feet of them; they broke in an instant and ran into the town, which was close by, and we after them, pell-mell. Many of them ran into a church, and we put a guard at the door to keep them there for the present, and some ran into the cellars of the houses.

We were then marched towards the market; when near it, General Washington came up on horse-back and alone, to our major, and said, "march on, my brave fellows, after me!" and rode off. We passed by a number of dead and wounded Hessians, and at length arrived at the other side of the town, where we beheld five or six hundred Hessians, who, it appeared, had been taken prisoners by our army, before our regiments arrived, that morning. We were ordered to guard them down to the ferry and conduct them across the river. About nine hundred Hessians were taken prisoners this day, all Grenadiers; I saw between three and four hundred of them, who had contrived to escape, but how they managed it, I was at a loss to discover. While we were marching to the ferry, the Hessians, seeing some of our men with the Hessian brass caps on, which they had taken from the dead, voluntarily gave us their caps and put on their hats, which they had with them; at this, our men were much pleased, and it was laughable to see how ill the brass caps comported with the rest of their habiliments, they being all in a pitiable condition for the want of decent or comfortable clothing, and some even without shoes to their feet.

The next day after the Battle of Trenton, being two days after the expiration of the term of our enlistment, we received three months' pay. Twenty-six dollars was offered to every man who would remain with the army only six weeks longer. For my part, being worn out with fatigue and sickness, I had made up my mind to quit the soldier's life, notwithstanding the earnest solicitations of the officers, who told me I was the life and soul of the company, and that I was to be promoted to an ensign if I remained with them. I therefore, in company with a boy, (also a fifer,) took my leave of them and set out on my return to my parents in Boston.

